# Reconstruction of gene regulatory modules from RNA silencing of IFN-α modulators: experimental set-up and inference method

**DOI:** 10.1186/s12864-016-2525-5

**Published:** 2016-03-12

**Authors:** Angela Grassi, Barbara Di Camillo, Francesco Ciccarese, Valentina Agnusdei, Paola Zanovello, Alberto Amadori, Lorenzo Finesso, Stefano Indraccolo, Gianna Maria Toffolo

**Affiliations:** Department of Surgery, Oncology and Gastroenterology, University of Padova, via Gattamelata 64, 35128 Padova, Italy; Department of Information Engineering, University of Padova, via Gradenigo 6/B, 35131 Padova, Italy; Istituto Oncologico Veneto - IRCCS, via Gattamelata 64, 35128 Padova, Italy; Institute of Electronics, Computer and Telecommunication Engineering, CNR, via Gradenigo 6/B, 35131 Padova, Italy; Present address: Department of Molecular Medicine, University of Padova, via Gabelli 63, 35121 Padova, Italy

**Keywords:** IFN-α modulators, Gene regulatory modules, RNA silencing, Experimental set-up, Small-scale gene expression screens

## Abstract

**Background:**

Inference of gene regulation from expression data may help to unravel regulatory mechanisms involved in complex diseases or in the action of specific drugs. A challenging task for many researchers working in the field of systems biology is to build up an experiment with a limited budget and produce a dataset suitable to reconstruct putative regulatory modules worth of biological validation.

**Results:**

Here, we focus on small-scale gene expression screens and we introduce a novel experimental set-up and a customized method of analysis to make inference on regulatory modules starting from genetic perturbation data, e.g. knockdown and overexpression data. To illustrate the utility of our strategy, it was applied to produce and analyze a dataset of quantitative real-time RT-PCR data, in which interferon-α (IFN-α) transcriptional response in endothelial cells is investigated by RNA silencing of two candidate IFN-α modulators, STAT1 and IFIH1. A putative regulatory module was reconstructed by our method, revealing an intriguing feed-forward loop, in which STAT1 regulates IFIH1 and they both negatively regulate IFNAR1. STAT1 regulation on IFNAR1 was object of experimental validation at the protein level.

**Conclusions:**

Detailed description of the experimental set-up and of the analysis procedure is reported, with the intent to be of inspiration for other scientists who want to realize similar experiments to reconstruct gene regulatory modules starting from perturbations of possible regulators. Application of our approach to the study of IFN-α transcriptional response modulators in endothelial cells has led to many interesting novel findings and new biological hypotheses worth of validation.

**Electronic supplementary material:**

The online version of this article (doi:10.1186/s12864-016-2525-5) contains supplementary material, which is available to authorized users.

## Background

One of the most discussed topics in the field of systems biology is the inference of gene regulatory networks (GRNs) from high-throughput expression data. Biological networks are graphical representations of the complex dependencies between the different molecular species interacting in a cell, i.e. genes, transcripts, proteins and metabolites, where nodes represent biological molecules and edges interactions. More precisely, GRNs aim to identify regulation at the transcript level, thus nodes represent genes and edges direct as well as indirect influences between genes. Network inference may be useful to elicit important regulatory mechanisms characterizing biological systems or involved, for instance, in the development of a complex disease, or in the action of a drug. Although, in the past decades, many different approaches have been proposed to infer gene regulation on a genome-wide scale [1–4], the reverse-engineering of GRNs still remains an open challenge, mainly due to the identifiability issues arising when working in the context of a limited number of available measurements compared with a huge number of genes [5].

Critical assessment of different inference methods has demonstrated that the most informative data come from multiple input experiments [6]. Systematic perturbation data were successfully used also in other contests, e.g. to construct quantitative models of signalling networks for predicting the effects of drug combinations [7]. As regards GRNs, genetic perturbations, in which the expression levels of one or more genes are altered by their silencing (knockout, knockdown) or up-regulation (overexpression), are the best suited to reconstruct gene regulatory relationships that account for directionality [4, 8]. Outstanding large-scale studies, in which different types of high-throughput data are integrated to provide a comprehensive view of the underlying transcriptional network, have been conducted (e.g. [9]). However, given the complexity and the high costs related to a whole-genome approach, it is a common practice to focus the attention on smaller regulatory sub-networks and on the basic building modules of which they are composed [10, 11].

Recurring interaction motifs have been shown to characterize cellular networks [12, 13] and, among them, the feed-forward loop (FFL) is particularly interesting for its properties. It is a closed unidirectional loop, composed by an upstream regulator X which controls a downstream regulator Y, and they both control a common target Z. Thus the regulation of X on Z is due to the balance of two effects: one direct and one mediated by Y. Among the three-node patterns, the FFL was the only one found to be a statistically significant motif in real transcription networks [14]. Moreover, it is an important functional circuit, whose dynamical properties make it suitable for the functions of noise suppression and adaptation [15].

In this paper we tackle the problem of inferring gene regulation in small sub-networks from both the experimental design perspective and the analysis pipeline used for regulatory motifs reconstruction. Specifically, our contribution addresses three main questions: (a) how to build up an experiment to investigate the transcriptional modulators of a biological process, optimizing the trade-off between costs and informative content of the data; (b) how to extract information inherent in perturbation data and (c) how to reconstruct putative FFL regulatory motifs, to be subsequently biologically validated.

We first describe our method in general terms, and then exemplify its ability to generate biological hypotheses on candidate modulators through a specific case study on interferon-α (IFN-α) transcriptional response in endothelial cells.

## Methods

Consider a biological system for which a transcriptional signature is available, from a genome-wide expression study. The purpose is to focus on a few candidate transcriptional modulators, and design a new experiment able to infer putative functional regulatory modules involving them. In the following, we first describe some crucial aspects related with the experimental set-up, to be addressed in order to provide an appropriate set of data. Then, the analysis pipeline will be presented, consisting of two main steps: a significance analysis to elicit significant modulations due to the silencing of each target gene, and an inference procedure to extract regulatory modules, combining the significant regulations induced by different couples of target genes. Although the selection procedure presented below is suited for the analysis of quantitative real-time RT-PCR (qRT-PCR) data, as in our case study, our method for inferring regulatory modules may be applied to different types of gene expression data, in cascade to any standard strategy of selection.

### Experimental set-up and pre-processing

#### Pre-selection of transcripts

To define the experimental set-up, the first crucial step is the pre-selection of the panel of genes to be monitored. A reasonable approach is to pool the following sets of genes:

- genes belonging to signalling pathways of interest;
- the most differentially expressed genes in the genome-wide signature, based on previous experiments;
- other genes of biological interest;
- few candidate housekeeping genes.

#### Single-gene perturbations

In order to reconstruct causal relationships between the perturbed gene and its targets, the expression of the panel of genes is monitored during perturbations of single candidate transcriptional modulators. Informative perturbation data include knockdown, knockout or overexpression.

In the next paragraph we will treat specifically the case of RNAi-mediated knockdown of candidate modulators.

#### Multiple-stimulation data

**Fig. 1:**
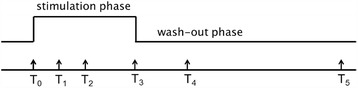
Example of sampling scheme. Data are collected at different time points during treatment and removal of a drug/agent, corresponding to stimulation and wash-out phase. Time points T_3_ and T_5_ are scheduled to monitor steady state gene expression in the stimulation and wash-out phase, respectively

Following each perturbation, samples are collected in short time-series, to capture events occurring at different time points, and, possibly, subjected to different stimuli, including treatment with a drug/agent, removal of the drug/agent, stimulation with a drug/agent at different doses. The sampling schedule should be designed in order to catch the dynamics induced by each perturbation, also based on a priori information, evaluating the trade-off between informative content and biological complexity of the experiment. An example of a sampling scheme with a double stimulation and a short time series is reported in Fig. 1.

#### Data pre-processing

Expression of preselected genes is quantified by multi-well plates containing custom qRT-PCR assays, under different gene perturbation conditions. Ready custom cards, with possibility of pre-selecting the spotted genes, are available from different companies and in several formats, with number of assays varying among 48, 64, 96, 192, 384. qRT-PCR data are normalized with respect to a reference gene *R*, shown to be stably expressed across the different conditions, and the expression level of each gene of interest is calculated via the comparative cycle threshold (*CT*) method [16]. The effects of each perturbation *P* targeting a specific gene *X* are thus evaluated with respect to a calibrator condition *C*, as ∆∆*CT* = ∆*CT*^*P*^–∆*CT*^C^ where ∆*CT* = *CT*_*X*_–*CT*_*R*_.

### Significance analysis

In this paragraph we treat the specific case of perturbations induced by siRNA knockdowns, with the intent of explicitly clarify the analysis pipeline adopted in the case study. To extract information from knockdown data about significant modulations that may represent direct or indirect effects due to the inactivation of target genes, a selection procedure, based on a measurement error model of biological variability, was devised. The proposed strategy was inspired by previous methods for robust quantization of differentially expressed genes in microarray data [17].

#### Measurement error model of *σ*_*ΔΔCT*_^2^

The biological variability of ∆∆*CT* is estimated from replicates through a flexible model for error variance,

1 $$ {\widehat{\sigma}}_{\varDelta \varDelta CT}^2=\alpha +\beta \cdot {\left|\varDelta \varDelta CT\right|}^{\gamma }, $$

where *α*, *β* and *γ* are parameters, linking the variance to the absolute value of the observed ∆∆*CT* intensities.

#### Selection procedure

We propose a two-stage approach that first filters observations by a variance based criterion and then performs a variable-by-variable statistical test procedure, that uses the biological variance estimated from the error model to assign a p-value to each modulation. For each silencing experiment, starting from the mean ∆∆*CT* values (across biological replicates), the detailed selection procedure consists of the following steps.

1. * Filtering based on ∆∆CT variance distribution.*We filtered out all the ∆∆*CT* whose variance exceeded the 95-th percentile of the observed variance distribution.
2. * Statistical test procedure.* For each gene and each time point *t*_*i*_, we tested the null hypothesis, *H*_0_ : *ΔΔCT* = 0, namely no difference in the effects of a siRNA targeting a specific gene and of the calibrator siRNA. A gene at a given time point is considered not differentially expressed if the ∆∆*CT* is close to 0. Under *H*_0_, the test statistic, i.e. the averaged ∆∆*CT*, was assumed to be distributed as a $N\left(0,{\widehat{\sigma}}^2/K\right)$, where *K* is the number of independent biological replicates and ${\widehat{\sigma}}^2$ is the biological variance of ∆∆*CT*, estimated by the measurement error model. The statistical test procedure results in a vector of p-values, one for each gene and time point.
3. * Multiple testing correction.* A Bonferroni multiple test correction is applied to control the false positive rate (FPR) in the gene callings. Significant modulations are defined by fixing a cut-off of 0.05 on the Bonferroni corrected *p*-values.

### Inference of multi-output FFL regulatory modules

Starting from the results of the selection procedure, i.e. from the Bonferroni corrected significant modulations induced by each silenced gene, we infer regulatory modules. The rationale is to extract, among significant regulations, those representing FFLs. The strategy is the following.

1. Select the couples of perturbed genes (P1, P2) for which regulation of P1 on P2 or vice versa is significant.
2. Identify the lists (L1, L2) of genes differentially regulated by P1 and P2, respectively.
3. Check whether L1 and L2 have genes in common.
4. For each shared modulation found, reconstruct the corresponding FFL. The type of regulation for each edge in the FFL is determined by the correlation between the expression levels of the genes at its ends. If a silenced gene significantly down-regulates (up-regulates) another gene, we interpret the regulation as an activation (repression) relationship, from the silenced gene to its target.
5. Merge the FFLs together to obtain a completely connected multi-output FFL regulatory module, formed by P1, P2 and their common targets.

The multi-output FFLs thus inferred may have functional roles and represent distilled information on which experimental researchers can focus for further investigation. Moreover, the FFL circuit is conceptually easy to be validated using, for instance, combinatorial gene silencing and/or passing from the mRNA to the protein level.

## Case study: IFN-α induced regulatory modules

Our approach was applied to deepen the knowledge gained from a recent genome-wide study in which the signature of IFN-α in human umbilical vein endothelial cells (HUVECs) was inspected after 5 h from stimulation, determining the significant up-regulation of 242 probesets [18]. Interferon-α is a cytokine endowed with antiviral, immunomodulatory and antiproliferative activities, achieved through the induction of hundreds of interferon-stimulated genes (ISGs). Discovered in 1957 [19] as a molecule capable of restricting viral replication in vivo, IFN-α has widely been used in the treatment of hepatitis C virus (HCV) infection as well as in some types of cancer, due to its pleiotropic and potent biological activities. The novel experimental set-up was used to investigate the role of few candidate modulators on IFN-α transcriptional response in endothelial cells. Here we analyze a subset of these data, specifically monitoring the transcriptional effects caused by the silencing of STAT1 and IFIH1, two known regulators of IFN-a signalling [20]. We decided to perturb a TF (STAT1) and a non-TF (IFIH1), to exemplify, in a real situation, the information that may be obtained by our procedure for both types of modulators.

### Materials and methods

Primary endothelial cells (HUVECs) were maintained in M200 culture medium supplemented with Low-Serum Growth Supplement (LSGS; Life Technologies, Paisley, UK) and stimulated in vitro with human recombinant IFN-α2 (Merck & Co., White House Station, NJ, USA) at the final concentration of 1000 IU/ml. Each biological sample was obtained by pooling cells from four different donors, at passage between 2 and 6. Stealth siRNA (Life Technologies) were used for RNAi-mediated knockdown of few candidate IFN-α modulators, including STAT1 and IFIH1 (catalogue IDs HSS110273 and HSS127414, respectively). Negative control with low GC content (Life Technologies) was used as calibrator siRNA. Full details on RNA silencing procedures are included in the Additional file 1.

**Fig. 2:**
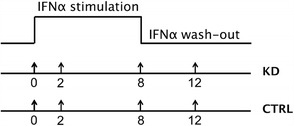
Sampling schedule adopted in the case study. Data are collected at 4 time points (0 h, 2 h, 8 h, 12 h), both during treatment and after removal of IFN-α, to monitor stimulation and wash-out phases. Two conditions are compared: samples with siRNA knockdown of the target gene (KD) and samples treated with the calibrator siRNA (CTRL)

The experiments were performed in biological duplicates. Samples were collected in short time series composed of 4 time points, with a double stimulation: IFN-α at 0 h and wash-out at 8 h, according to the sampling scheme in Fig. 2. The rationale underlying this sampling scheme was to test whether the candidate modulators exerted their action in the early (2 h) or/and late (8 h) IFN-α activation phase or/and in the phase of IFN-α removal (12 h). A panel of 96 pre-selected genes, related to IFN-α transcriptional response, was screened by Custom TaqMan Array Cards (Life Technologies, format 96b), under different gene perturbation conditions: siRNA inactivation of target genes. Transcripts to be monitored on the custom cards (Additional file 1: Table S1) were chosen as follows:

- 9 genes from the IFN-α signaling pathway;
- 75 genes from the top of the transcriptional signature found in [18], with respect to the fold change ranking;
- 9 genes of biological interest expressed in endothelial cells;
- 3 candidate housekeeping genes.

The qRT-PCR amplifications were run on an ABI Prism 7900HT Sequence Detection System (Life Technologies). Raw data were extracted using the SDS v2.4 software package (Life Technologies). All the following analyses were executed by developing custom code in the R statistical environment. JAK1 was shown to be the most stable gene across the different conditions and it was taken as reference gene for the normalization of qRT-PCR data and the calculation of ∆∆*CT*.

Methods used for experimental validation are described in the Additional file 1.

## Results

### Modelling of the ∆∆*CT* measurement error

**Fig. 3:**
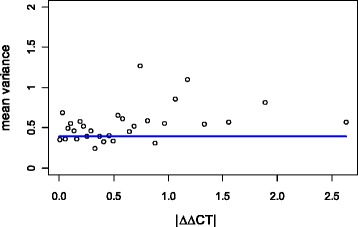
∆∆*CT* measurement error model. Absolute ∆∆*CT* intensities are binned, and, for each bin, the mean variance estimates are plotted against the mean |ΔΔ*CT*| intensities. The fitted model variance is ${\widehat{\sigma}}^2= const=0.3975$

After calculation of ∆∆*CT*, the biological variance was estimated via the general measurement error model in equation (1). The best fitting, in terms of Weighted Residual Sum of Squares (WRSS) and precision of the parameter estimates, was obtained by the model ${\widehat{\sigma}}^2=0.3975$ (Fig. 3). This estimate was based on the biological replicates related to the silencing of five candidate IFN-α modulators, including STAT1 and IFIH1.

### Genes significantly regulated by RNA silencing of candidate IFN-α modulators

**Fig. 4:**
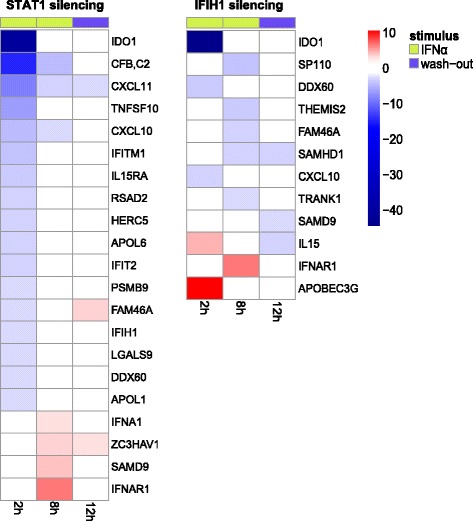
Heatmaps with the genes significantly regulated by STAT1 (left) and IFIH1 silencing (right) during the two phases: stimulation with IFN-α (2 h, 8 h) and 4 h after its removal (12 h). Colored tiles indicate significant regulations, down-regulation in blue and up-regulation in red. The intensities represent fold change (FC) inductions according to the color legend on side. Genes, from top to bottom, are ordered by increasing FC in the stimulation phase

The selection procedure led to the characterization of the significant regulations induced by the inactivation of each of the two candidate IFN-α modulators, STAT1 and IFIH1. Each modulator was thus evaluated both for its strength (number of genes significantly regulated following its silencing), its sign (positive, whether its inactivation mainly down-regulates the monitored genes or negative, otherwise) and the timing at which it exerts its prevalent action: early (2 h) or late (8 h) IFN-α stimulation phase or IFN-α removal phase (12 h). Results are presented as heatmaps in Fig. 4. A total of 21 and 12 genes were found to be significantly regulated by STAT1 and IFIH1, respectively. As expected, STAT1, a transcription factor central to IFN-α pathway, was here confirmed as a strong positive IFN-α modulator, with 17/21 genes down-regulated in the early stimulation phase, whereas IFIH1 was seen to be mainly a positive modulator with 8/12 down-regulated genes, 3 in the early and 5 in the late stimulation phase. Both modulators were shown to act in presence of IFN-α stimulus; the unique exception being the IFIH1 action exerted on SAMD9 only during the wash-out phase. Detailed information on statistical significance and FC of each regulation is reported in Additional file 1: Tables S2 and S3.

### STAT1 transcriptional regulations with validated binding sites

The whole set of STAT1 transcriptional regulations in *Homo sapiens* was extracted from the TRANSFAC Professional database (BIOBASE, release 2013.3), a manually curated database of eukaryotic transcription factors (TFs), their genomic binding sites and DNA binding profiles [21]. The targets of STAT1, identified by our analysis, were thus compared with the validated targets present in TRANSFAC, to check if some of the significant regulations found by our method were confirmed as direct regulations, having the target gene an already validated binding site. The results are summarized in Table 1. Interestingly, both IDO1, the gene found most importantly down-regulated by our significance analysis, and PSMB9 have validated binding sites for STAT1, thus confirming 2 out of the 21 significant regulations induced by STAT1. It is worth noting that among the direct targets of STAT1 with experimentally validated binding sites there are several interferon regulatory factors (IRF1, IRF7, IRF8) and also other genes implicated in the IFN response (e.g. GBP1). Considering also indirect targets, i.e. targets mediated by TFs regulated by STAT1, other two significant regulations are confirmed as indirect, having CXCL10 and IFITM1 experimentally validated binding sites for IRF1. Overall, the analysis on the TRANSFAC Professional database confirms 4 out of the 21 significant regulations induced by STAT1. Two of them are direct regulations and two indirect ones, possibly mediated by IRF1. The presence of a direct regulation between STAT1 and IFIH1 is supported by a recent paper, demonstrating, by using ChIP-chip data and ChIP-PCR validation on independent biological samples, that phosphorylated STAT1 binding site is present in the IFIH1 promoter [22].

**Table 1:** STAT1 targets with validated binding sites out of the 21 inferred putative targets

Transcription Factor	Target	Source
STAT1	IDO1	TRANSFAC release 2013.3
STAT1	PSMB9	TRANSFAC release 2013.3
STAT1	IFIH1	Przanowski et al. [22]
STAT1	IRF1	TRANSFAC release 2013.3
IRF1	CXCL10	TRANSFAC release 2013.3
IRF1	IFITM1	TRANSFAC release 2013.3

Columns indicate the transcription factor, its validated target and the source from which the validation is extracted, respectively. Direct targets of STAT1 are reported in the first three rows, whereas indirect targets, mediated by IRF1, are shown in the last two rows

### Reconstruction of regulatory modules involving IFN-α modulators

**Fig. 5:**
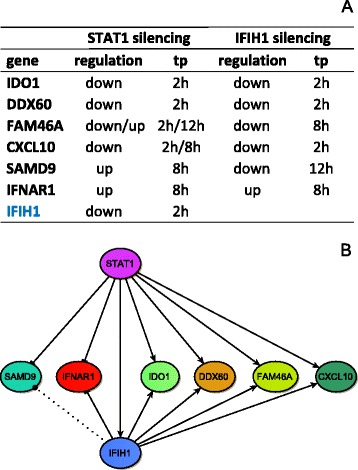
Inference of putative regulatory modules. **a** A summary of genes significantly modulated by STAT1 and IFIH1 silencing, extracted from Fig. 4. Since IFIH1 was significantly down-regulated by STAT1 silencing, each gene reported in the table is the common target of a FFL motif involving STAT1 and IFIH1. **b** The inferred six-output FFL regulatory module. Lines represent influence regulations in the stimulation (2 h/8 h; solid line) and wash-out (12 h; dashed line) phase. Arrow styles stand for: arrow, activation; ⊣, repression; dot, unspecified sign of regulation

Combining the significant modulations due to the silencing of different modulators, our approach allows to reconstruct putative regulatory modules that provide distilled information worth of biological validation. Fig. 5 illustrates how the procedure is able to reconstruct the regulatory sub-network involving two candidate IFN-α modulators, STAT1 and IFIH1. First, a connection is suggested between the two nodes representing them, since IFIH1 was significantly regulated by STAT1. By looking at their common targets, IDO1, DDX60, FAM46A, CXCL10, SAMD9, and IFNAR1 appear to be significantly regulated by both. Each shared regulation correspond to a FFL including STAT1, IFIH1 and one common target. Merging together the six FFLs, the regulatory module shown in Fig. 5b is inferred. Regulations occurring in the IFN-α removal phase (at 12 h in our set-up) are graphically represented as dotted lines, without specifying whether they indicate activations or repressions. If a gene is regulated both in the stimulation and wash-out phase, e.g. FAM46A, only the regulation in the stimulation phase is reported in the reconstructed network. All the edges in the network represent influence relationships between pairs of genes, that may correspond to direct (due to the binding of the regulator to the promoter region of its target), as well indirect (e.g. mediated by other factors), transcriptional regulations. In particular we expect that some of the regulations induced by STAT1, which is a TF, are direct as those on IDO1 and IFIH1 and others indirect, e.g. that of STAT1 on CXCL10, probably due to a transcription factor regulatory pathway mediated by IRF1 (see previous paragraph). The influence relationships induced by IFIH1, which is not a TF, are probably indirect and more difficult to be interpreted but are still useful to elicit its role of IFN-α transcriptional modulator.

### STAT1 is a negative regulator of IFNAR1

**Fig. 6:**
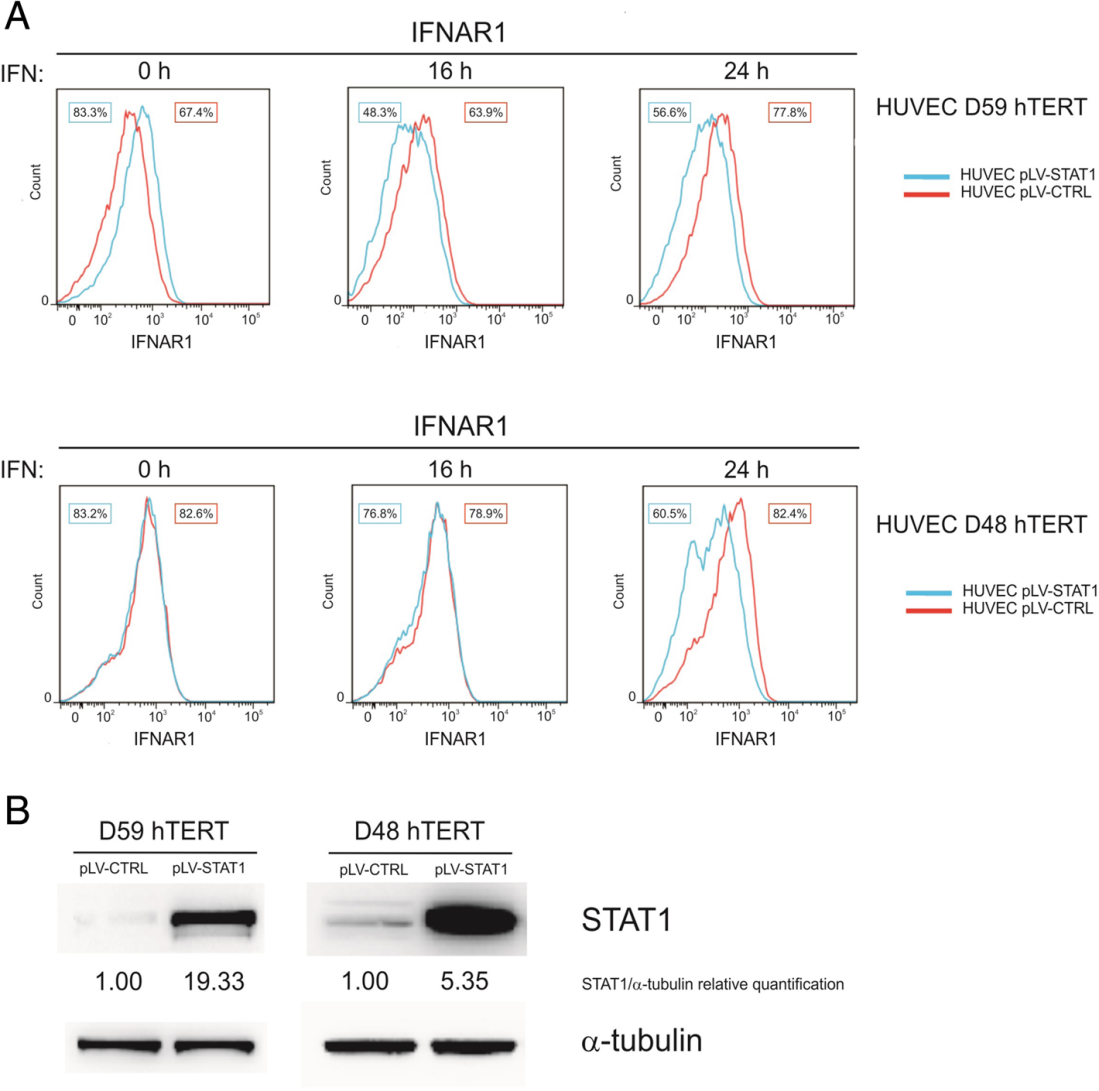
Validation of STAT1 negative regulation on IFNAR1. **a** Flow cytometric analysis of HUVEC cells transduced with a lentiviral vector coding wild-type STAT1 cDNA. STAT1 overexpression caused down-regulation of surface IFNAR1 expression after 16 h (D59 hTERT) and 24 h (both donors) of IFN-α stimulation in two different endothelial cell cultures (HUVEC D48 and D59 hTERT). Percentage of positive cells is reported for both HUVECs transduced with pLV-STAT1 and pLV-CTRL. IFNAR1 expression was evaluated relative to isotype control (not shown). **b** Western blot analysis of STAT1 levels in HUVECs used in (**a**). α-tubulin hybridization is shown as a loading control. For quantification of STAT1 expression, density values were normalized to the STAT1/α-tubulin ratio measured in the control sample (pLV-CTRL)

Among the six putative FFLs (Fig. 5b), the one in which STAT1 regulates IFIH1 and they both regulate IFN-α receptor subunit 1 (IFNAR1) is particularly interesting because it seems to indicate the presence of a negative feedback on an element upstream in the IFN-α pathway, the receptor itself. Thus, STAT1 negative regulation on IFNAR1 was object of experimental validation. As a first attempt, we tried to replicate the STAT1 silencing effects at the protein level but in three different donors we did not detect increased IFNAR1 levels following siRNA-mediated silencing of STAT1 (data not shown). This behaviour was possibly due to the high baseline levels of IFNAR1 expression in HUVECs, confirmed also in additional six samples. We thus opted for the complementary approach. To investigate effects of STAT1 overexpression on IFNAR1, HUVECs were transduced with STAT1-encoding (LV-STAT1) or control (LV-CTRL) lentiviral vectors and analyzed by flow cytometry following IFN-α stimulation. In two different HUVEC samples (D48 and D59) we observed that although STAT1 overexpression did not apparently modulate IFNAR1 surface levels in unstimulated cells (Fig. 6a, 0 h), STAT1 overexpression led to down-regulation of IFNAR1 in IFN-α stimulated cells (Fig. 6a). Western blot analysis confirmed that genetic modulation of STAT1 levels was followed by corresponding modulations in STAT1 protein levels (Fig. 6b). In order to exclude the possibility that the method adopted to overexpress STAT1 caused downregulation in the expression of other surface proteins, relevant control was performed on CD31, a structural protein normally present in endothelial cells. Following STAT1 overexpression, a mild down-regulation in the intensity of CD31 was observed but, differently from what was seen for IFNAR1, it did not involve the percentage of positive cells and it was present also in absence of IFN-α stimulus (Additional file 1: Figure S1).

These results indicate that STAT1 is able to negatively modulate IFNAR1 expression on cell membrane, thus confirming one of the putative regulation in the inferred FFL and revealing an unprecedented negative feedback loop in the IFN-α signalling pathway.

## Discussion

In this paper, we address to small-scale gene expression screens and present an original experimental set-up and a customized method of analysis suited to infer putative regulatory modules, extracting information inherent in genetic perturbation data. Our approach, result of the joint efforts of experimental and theoretical researchers, is applied to investigate the role of STAT1 and IFIH1 as candidate modulators of IFN-α transcriptional response in endothelial cells. The case study is used to exemplify the various steps of our approach and its ability to generate potentially biologically meaningful gene modules.

### Experimental set-up and analysis method

In the past decades, with the advent of microarrays, a huge amount of transcriptional studies have been conducted, but most of them were meant to identify transcriptional signatures, by taking a snapshot of differentially expressed genes at a fixed time point. Our experimental and computational framework, in its entirety, is thought to be applied in cascade to such studies and used to identify key regulators and the regulatory modules in which they participate.

In our experimental set-up, we propose to screen a panel of preselected genes under single-gene perturbations of candidate modulators by multi-well plates containing custom qRT-PCR assays. A set-up that combines RNA silencing with qRT-PCR data was already used in [23] to silence transcription factors (TFs) and create a TF regulatory sub-network in hepatoma cells, considering the significant modulations found among eight hepatocyte human factors. However, our experimental framework has a more general applicability, as we describe in detail how to design a new biological experiment, give hints about the pre-selection of the transcripts to be monitored and suggest an informative sampling scheme, including multiple stimuli and a time series. Besides, we describe the computational method used to elicit significant regulations induced by each perturbation (significance analysis) and to reconstruct putative multi-output FFL regulatory modules (inference method). Finally, while in [23] the focus was only on TFs, in the case study we show that our approach is applicable also to genes that are not transcription factors (non-TFs). In fact, several examples are available in the literature of non-TFs able to modulate specific transcriptional networks [24, 25]. As far as IFN-α response is concerned, a prominent example is the modulation of JAK-STAT signalling by the ubiquitin peptidase USP18 [26, 27].

The proposed inference method is straightforward and much less computational demanding with respect to the wide number of existing methods for gene network reconstruction [4, 7]. Although exemplified on qRT-PCR data, the inference method can be easily extended to other types of gene expression data, e.g. may be applied to a microarray dataset processed with any standard significance analysis procedure to distil putative regulatory sub-networks or selected FFLs for experimental validation.

The major limitation of our approach is that, since we are not monitoring the whole genome, the regulations inferred are not necessarily direct, but may be mediated by genes that are not present in the custom array. The edges reconstructed in the putative regulatory modules, thus, represent influence relationships that may be confirmed or not by biological validation.

From a systems biology perspective, the multi-output FFLs, inferred by perturbing different couples of modulators that share target genes, may be merged to reconstruct a completely connected regulatory sub-network in which the perturbed genes are the only regulators. Such information could help in developing new mechanistic hypotheses and design new biological studies, e.g. allowing to identify sentinel genes that are responsive to multiple perturbations applied to the process under study.

### Case study: IFN-α induced regulatory modules

The application of our framework to construct and analyze a dataset of qRT-PCR data to assess the role played by STAT1 and IFIH1 on IFN-α transcriptional response in endothelial cells produced new biological insight. RNAi-mediated knockdown of STAT1 and IFIH1 was achieved by using target-specific stealth siRNAs. Genes monitored in custom qRT-PCR assays were preselected by taking the genes in IFN-α signalling pathway, as well as the genes most strongly modulated in the static IFN-α signature, in order to have an effective instrument to test the integrity of IFN-α transcriptional response. Given the difficulty to obtain adequate numbers of primary HUVEC samples and to get enough cells at a passage lower than 6, we performed two biological replicates in short time series. Two stimuli were applied: IFN-α at 0 h and its removal at 8 h, allowing to monitor two time points in the early (2 h) and late (8 h) IFN-α stimulation phase and one in the wash-out phase (12 h). The devised sampling scheme was useful to observe that both STAT1 and IFIH1 prevalently act as modulators in the activation phase, when the stimulation is present. STAT1, in particular, seems to have no more effects on the panel of selected genes four hours after IFN-α removal (time point 12 h in the wash-out phase). This observation suggests that STAT1 acts as modulator only in presence of the stimulus, consistently with flow cytometric analysis in Fig. 6, showing that overexpression of STAT1 does not cause down-regulation on IFNAR1 in absence of IFN-α.

From our analysis, STAT1 shows to act as early positive IFN-α modulator, indeed its inactivation leads to the down-regulation of 17 genes, already 2 h after IFN-α stimulation. The four genes (IFNA1, ZC3HAV1, SAMD9 and IFNAR1) that appear to be negatively regulated by STAT1 are up-regulated 8 h after IFN-α stimulation. This phenomenon might reflect the involvement of some early negative regulator that is induced by STAT1 silencing and/or IFN-α stimulation. The effects of IFIH1 silencing on the expression of genes tested appear more complicated. At first glance, the modulation of the IFN-α transcriptional network induced by IFIH1 perturbation could seem counterintuitive, due to its non-TF nature. However, it has been demonstrated that IFIH1 controls activation of a transcription factor (IRF3) in Enterovirus-infected cells [28]. Therefore, it can be speculated that a similar mechanism could be at play in our model. Another hypothesis is that the action of IFIH1 on its targets could be miRNA mediated. Indeed, IFIH1 RNA helicase domain could be involved in miRNA processing, as described for other RNA helicases and DEAD box-containing proteins [29, 30].

An interesting interplay was discovered, being STAT1 a regulator of IFIH1 and sharing the two six target genes, as depicted in the putative six-output FFL of Fig. 5b. A discovered FFLs was particularly intriguing, namely the one showing that STAT1 positively regulates IFIH1 and they both negatively regulates the expression of IFN-α receptor subunit 1. IFN-α signalling pathway is triggered by IFN-α that binds to its cell-surface receptor IFNAR, consisting of two subunits IFNAR1 and IFNAR2. The former is the ligand-binding unit that, after interaction with IFN-α, recruits the signal transducer unit, IFNAR2. The binding of the ligand to IFNAR results in the cross-activation of two Janus protein tyrosine kinases (TYK2 and JAK1), which then phosphorylate their downstream substrates, STAT1 and STAT2. The latter interact with IRF9 to form a specific transcriptional activator complex, ISGF3, which results in the transcriptional induction of hundreds IFN-stimulated genes. Therefore, the finding that STAT1 negatively regulates IFNAR1, which has been biologically validated as part of this study (Fig. 6), could be an efficient regulatory mechanism to restrict signalling and biological response induced by IFN-α in endothelial cells.

## Conclusions

A novel experimental and computational framework for the design and analysis of gene expression experiments on small-scale and with limited budget was presented. Our method extracts information inherent in perturbation data to reconstruct putative multi-output FFL regulatory modules, thus generating potentially new biological hyphotheses.

Application to the study of IFN-α transcriptional response modulators in endothelial cells has led to many interesting novel findings and to the experimental validation of the negative regulation of STAT1, a TF central in IFN-α signalling pathway, on IFNAR1, the receptor itself. Further biological experiments are ongoing and will be presented in future studies where the role of other IFN-α modulators will be investigated.

## Additional file

Additional file 1: Supplementary Material including text, tables and figures. (PDF 115 kb)

## Abbreviations

IFN-α: interferon-α
GRNs: gene regulatory networks
FFL: feed-forward loop
qRT-PCR: quantitative real-time RT-PCR
CT: cycle threshold
HUVECs: human umbilical vein endothelial cells
TFs: transcription factors
