# Supplementary material for: Reconstruction of gene regulatory modules from RNA silencing of IFN-α modulators: experimental set-up and inference method
Source: BMC Genomics. 2016 Mar 12;17:228. doi: 10.1186/s12864-016-2525-5 (PMC4788926; doi:10.1186/s12864-016-2525-5)
Supplement: Supplementary file 1 — Supplementary Material including text, tables and figures. (PDF 115 kb) [file 12864_2016_2525_MOESM1_ESM.pdf]

SUPPLEMENTARY MATERIAL

RNA silencing procedures

Sequences of Stealth siRNAs are reported in SupplementaryTable S4.
HUVECs were plated (3,65x10^4/cm^2) on tissue culture plates and, the following day, were transfected using Lipofectamine RNAiMAX Transfection Reagent (Life Technologies). In detail, each siRNA was diluted in 500 µl Opti-MEM® I with GlutaMAX™-I transfection medium (Life Technologies) with 5 µl Lipofectamine RNAiMAX Transfection Reagent and added to 1.5 ml culture medium (M200). Final concentration of each siRNA was 10 nM. Transfection was blocked 6 hours post-siRNA administration by double washing with culture medium (RPMI) without serum. For each target gene a set of three siRNAs was tested for RNAi activity and the Stealth siRNA showing the highest level of knockdown (>75%) was selected to be used for subsequent experiments. IFN-α stimulation was carried out 48 hours post-transfection. RNA was extracted using phenol/chloroform method.
For all knockdown experiments, RNA quality was checked by agarose gel loading and by measurement of absorbance at 230 nm, 260 nm and 280 nm. Total RNA (2 µg) was reverse transcribed using High Capacity RNA-to-cDNA Kit (Life Technologies), according to manufacturer's instructions. Silencing of target genes was measured by SYBR® Green (Life Technologies) qRT-PCR using primers in Supplementary Table S5. Hydroxymethylbilane synthase (HMBS) was used as reference gene.

Methods used for experimental validation

STAT1 overexpression. STAT1 was overexpressed using pLV-STAT1 lentiviral vector. Total RNA (2 µg) was reverse transcribed using High Capacity RNA-to-cDNA Kit (Life Technologies), according to manufacturer's instructions.

Cytofluorimetric analysis. IFNAR1 expression on endothelial cells surface was evaluated using a rabbit anti-IFNAR1 primary antibody (Abcam, Cambridge, UK) and, subsequently, an anti-rabbit Alexa Fluor 488 secondary antibody (Life Technologies). CD31 expression was analysed using a FITC-labeled antibody (Coulter, Fullerton, CA). Data acquisition was performed with BD LSRII flow cytometer (Biosciences, San Jose, CA, USA) and data were analyzed with FlowJo software (TreeStar Inc).

Western blot analysis. Cell lysates were run on 4-12% polyacrylamide gels. Separated proteins were then blotted onto a nitrocellulose membrane for 2h at 400 mA. Immunoprobing was performed using rabbit polyclonal Ab against STAT1 (Thermo Fisher Scientific, Waltham, MA, USA) and mouse mAb against α-tubulin (Sigma-Aldrich, Saint Louis, MO, USA), followed by hybridization with horseradish peroxidase-conjugated anti-rabbit and anti-mouse Abs (Amersham-Pharmacia, Little Chalfont, UK), respectively. Antigens were identified by luminescent visualization using the Western Lightning® Plus-ECL Kit (PerkinElmer, Waltham, MA, USA).

Supplementary Tables

Table S1. List of genes monitored on Custom TaqMan Array Cards (Life Technologies, format 96b).

Assay ID	Gene Symbol	Gene Name
**Hs99999901_s1**	18S	Eukaryotic 18S rRNA
**Hs00222415_m1**	APOBEC3G	apolipoprotein B mRNA editing enzyme, catalytic polypeptide-like 3G
**Hs00358603_g1**	APOL1	apolipoprotein L, 1
**Hs01935263_s1**	APOL2	apolipoprotein L, 2
**Hs00600896_m1**	APOL3	apolipoprotein L, 3
**Hs00229051_m1**	APOL6	apolipoprotein L, 6
**Hs00231069_m1**	ATF3	activating transcription factor 3
**Hs00388703_m1**	BLZF1	basic leucine zipper nuclear factor 1
**Hs00969291_m1**	BNIP3	BCL2/adenovirus E1B 19kDa interacting protein 3
**Hs00171632_m1**	BST2	bone marrow stromal cell antigen 2
**Hs00251097_m1**	C19orf66	chromosome 19 open reading frame 66
**Hs00354836_m1**	CASP1	caspase 1, apoptosis-related cysteine peptidase (interleukin 1, beta, convertase)
**Hs00156060_m1**	CFB,C2	complement factor B,complement component 2
**Hs00171086_m1**	CX3CL1	chemokine (C-X3-C motif) ligand 1
**Hs00171042_m1**	CXCL10	chemokine (C-X-C motif) ligand 10
**Hs00171138_m1**	CXCL11	chemokine (C-X-C motif) ligand 11
**Hs00204833_m1**	DDX58	DEAD (Asp-Glu-Ala-Asp) box polypeptide 58
**Hs00214153_m1**	DDX60	DEAD (Asp-Glu-Ala-Asp) box polypeptide 60
**Hs00225561_m1**	DHX58	DEXH (Asp-Glu-X-His) box polypeptide 58
**Hs00189422_m1**	DSP	Desmoplakin
**Hs00152844_m1**	ELF1	E74-like factor 1 (ets domain transcription factor)
**Hs00214159_m1**	FAM46A	family with sequence similarity 46, member A
**Hs00266717_m1**	GBP1	guanylate binding protein 1, interferon-inducible, 67kDa
**Hs00609198_m1**	GCH1	GTP cyclohydrolase 1
**Hs00199328_m1**	GMPR	guanosine monophosphate reductase
**Hs00180943_m1**	HERC5	hect domain and RLD 5
**Hs00215555_m1**	HERC6	hect domain and RLD 6
**Hs00609296_g1**	HMBS	hydroxymethylbilane synthase
**Hs00158027_m1**	IDO1	indoleamine 2,3-dioxygenase 1
**Hs00986755_m1**	IFI16	interferon, gamma-inducible protein 16
**Hs00271467_m1**	IFI27	interferon, alpha-inducible protein 27
**Hs00173838_m1**	IFI30	interferon, gamma-inducible protein 30
**Hs00413458_m1**	IFI35	interferon-induced protein 35
**Hs00197427_m1**	IFI44	interferon-induced protein 44
**Hs00199115_m1**	IFI44L	interferon-induced protein 44-like
**Hs00242571_m1**	IFI6	interferon, alpha-inducible protein 6
**Hs00223420_m1**	IFIH1	interferon induced with helicase C domain 1
**Hs00356631_g1**	IFIT1	interferon-induced protein with tetratricopeptide repeats 1
**Hs00533665_m1**	IFIT2	interferon-induced protein with tetratricopeptide repeats 2
**Hs00155468_m1**	IFIT3	interferon-induced protein with tetratricopeptide repeats 3
**Hs00202721_m1**	IFIT5	interferon-induced protein with tetratricopeptide repeats 5
**Hs00705137_s1**	IFITM1	interferon induced transmembrane protein 1 (9-27)
**Hs00256882_s1**	IFNA1	interferon, alpha 1
**Hs01066118_m1**	IFNAR1	interferon (alpha, beta and omega) receptor 1
**Hs01022059_m1**	IFNAR2	interferon (alpha, beta and omega) receptor 2
**Hs02621180_s1**	IFNB1	interferon, beta 1, fibroblast
**Hs01003716_m1**	IL15	interleukin 15
**Hs00233692_m1**	IL15RA	interleukin 15 receptor, alpha

**Hs00971959_m1**	IRF1	interferon regulatory factor 1
**Hs01547283_m1**	IRF3	interferon regulatory factor 3
**Hs00242190_g1**	IRF7	interferon regulatory factor 7
**Hs00196051_m1**	IRF9	interferon regulatory factor 9
**Hs00192713_m1**	ISG15	ISG15 ubiquitin-like modifier
**Hs00158122_m1**	ISG20	interferon stimulated exonuclease gene 20kDa
**Hs00233820_m1**	JAK1	Janus kinase 1
**Hs00371321_m1**	LGALS9	lectin, galactoside-binding, soluble, 9
**Hs01108898_g1**	LMNA	lamin A/C
**Hs00895608_m1**	MX1	myxovirus (influenza virus) resistance 1, interferon-inducible protein p78 (mouse)
**Hs01550808_m1**	MX2	myxovirus (influenza virus) resistance 2 (mouse)
**Hs00973637_m1**	OAS1	2,5-oligoadenylate synthetase 1, 40/46kDa
**Hs00942643_m1**	OAS2	2-5-oligoadenylate synthetase 2, 69/71kDa
**Hs00196324_m1**	OAS3	2-5-oligoadenylate synthetase 3, 100kDa
**Hs00984390_m1**	OASL	2-5-oligoadenylate synthetase-like
**Hs00221227_m1**	PLEKHA4	pleckstrin homology domain containing, family A (phosphoinositide binding specific) member 4
**Hs00275514_m1**	PLSCR1	phospholipid scramblase 1
**Hs00231241_m1**	PML	promyelocytic leukemia
**Hs00544758_m1**	PSMB8	proteasome (prosome, macropain) subunit, beta type, 8 (large multifunctional peptidase 7)
**Hs00160610_m1**	PSMB9	proteasome (prosome, macropain) subunit, beta type, 9 (large multifunctional peptidase 2)
**Hs01058986_m1**	RARRES3	retinoic acid receptor responder (tazarotene induced) 3
**Hs00369813_m1**	RSAD2	radical S-adenosyl methionine domain containing 2
**Hs00223342_m1**	RTP4	receptor (chemosensory) transporter protein 4
**Hs00415836_m1**	SAMD9	sterile alpha motif domain containing 9
**Hs00210019_m1**	SAMHD1	SAM domain and HD domain 1
**Hs00356334_m1**	SECTM1	secreted and transmembrane 1
**Hs00275455_m1**	SLC15A3	solute carrier family 15, member 3
**Hs00258556_s1**	SLC25A28	solute carrier family 25, member 28
**Hs00185406_m1**	SP110	SP110 nuclear body protein
**Hs00234829_m1**	STAT1	signal transducer and activator of transcription 1, 91kDa
**Hs00237139_m1**	STAT2	signal transducer and activator of transcription 2, 113kDa
**Hs00184465_m1**	TAP1	transporter 1, ATP-binding cassette, sub-family B (MDR/TAP)
**Hs00241066_m1**	TAP2	transporter 2, ATP-binding cassette, sub-family B (MDR/TAP)
**Hs00380413_m1**	TDRD7	tudor domain containing 7
**Hs00191466_m1**	THEMIS2	thymocyte selection associated family member 2
**Hs00152933_m1**	TLR3	toll-like receptor 3
**Hs00251020_m1**	TMEM140	transmembrane protein 140
**Hs00921974_m1**	TNFSF10	tumor necrosis factor (ligand) superfamily, member 10
**Hs99999147_m1**	TP53	tumor protein p53
**Hs00322331_m1**	TRANK1	tetratricopeptide repeat and ankyrin repeat containing 1
**Hs00207650_m1**	TRIM14	tripartite motif-containing 14
**Hs00172616_m1**	TRIM21	tripartite motif-containing 21
**Hs00177464_m1**	TYK2	tyrosine kinase 2
**Hs00188450_m1**	UBE2L6	ubiquitin-conjugating enzyme E2L 6
**Hs00276441_m1**	USP18	ubiquitin specific peptidase 18
**Hs00173626_m1**	VEGFA	vascular endothelial growth factor A
**Hs00213882_m1**	XAF1	XIAP associated factor 1
**Hs00912657_m1**	ZC3HAV1	zinc finger CCCH-type, antiviral 1

Table S2. Genes significantly modulated by STAT1 silencing.

Gene Symbol - Assay ID	TP	p-value	adjusted-p	FC
IDO1-Hs00158027_m1	2h	6,49E-33	1,49E-31	-40,15
CFB,C2-Hs00156060_m1	2h	2,36E-18	1,08E-16	-14,89
CXCL11-Hs00171138_m1	2h	3,73E-12	8,59E-11	-8,56
TNFSF10-Hs00921974_m1	2h	8,27E-10	1,90E-08	-6,67
CXCL10-Hs00171042_m1	2h	2,15E-07	4,95E-06	-4,96
CFB,C2-Hs00156060_m1	8h	5,44E-07	2,50E-05	-4,70
STAT1-Hs00234829_m1	12h	1,91E-06	4,39E-05	-4,36
IFITM1-Hs00705137_s1	2h	2,98E-06	6,85E-05	-4,24
IL15RA-Hs00233692_m1	2h	4,95E-05	0,001	-3,50
RSAD2-Hs00369813_m1	2h	5,65E-05	0,002	-3,47
STAT1-Hs00234829_m1	8h	1,50E-04	0,003	-3,23
HERC5-Hs00180943_m1	2h	1,50E-04	0,003	-3,23
APOL6-Hs00229051_m1	2h	2,28E-04	0,005	-3,12
CXCL11-Hs00171138_m1	8h	2,31E-04	0,005	-3,12
STAT1-Hs00234829_m1	2h	2,66E-04	0,006	-3,09
IFIT2-Hs00533665_m1	2h	2,67E-04	0,006	-3,08
CXCL10-Hs00171042_m1	8h	5,89E-04	0,014	-2,89
PSMB9-Hs00160610_m1	2h	6,13E-04	0,014	-2,88
FAM46A-Hs00214159_m1	2h	8,97E-04	0,021	-2,79
IFIH1-Hs00223420_m1	2h	9,12E-04	0,021	-2,79
LGALS9-Hs00371321_m1	2h	1,02E-03	0,023	-2,76
CXCL11-Hs00171138_m1	12h	1,65E-03	0,038	-2,64
DDX60-Hs00214153_m1	2h	1,69E-03	0,052	-2,64
APOL1-Hs00358603_g1	2h	1,92E-03	0,044	-2,61
ZC3HAV1-Hs00912657_m1	12h	1,18E-03	0,027	2,72
IFNA1-Hs00256882_s1	8h	5,14E-04	0,012	2,93
ZC3HAV1-Hs00912657_m1	8h	2,79E-04	0,006	3,07
FAM46A-Hs00214159_m1	12h	1,24E-04	0,003	3,27
SAMD9-Hs00415836_m1	8h	2,94E-05	0,001	3,64
IFNAR1-Hs01066118_m1	8h	2,22E-09	5,10E-08	6,35

Table S3. Genes significantly modulated by IFIH1 silencing.

Gene Symbol - Assay ID	TP	p-value	adjusted-p	FC
IDO1-Hs00158027_m1	2h	1,26E-34	3,83E-33	-44,37
IFIH1-Hs00223420_m1	8h	7,33E-10	1,67E-08	-6,71
IFIH1-Hs00223420_m1	12h	4,32E-07	9,83E-06	-4,77
SP110-Hs00185406_m1	8h	1,97E-06	5,98E-05	-4,35
IFIH1-Hs00223420_m1	2h	1,21E-05	2,74E-04	-3,87
DDX60-Hs00214153_m1	2h	2,08E-05	4,72E-04	-3,73
THEMIS2-Hs00191466_m1	8h	3,64E-05	0,001	-3,58
IL15-Hs01003716_m1	12h	5,72E-05	0,003	-3,47
FAM46A-Hs00214159_m1	8h	9,42E-05	0,003	-3,34
SAMHD1-Hs00210019_m1	8h	1,98E-04	0,005	-3,16
SAMHD1-Hs00210019_m1	12h	2,16E-04	0,005	-3,14
CXCL10-Hs00171042_m1	2h	2,71E-04	0,008	-3,08
SAMD9-Hs00415836_m1	12h	8,06E-04	0,024	-2,82
TRANK1-Hs00322331_m1	8h	1,61E-03	0,037	-2,65
IL15-Hs01003716_m1	2h	1,61E-06	7,30E-05	4,40
IFNAR1-Hs01066118_m1	8h	1,87E-09	5,66E-08	6,40
APOBEC3G-Hs00222415_m1	2h	2,86E-14	8,67E-13	10,48

Table S4. Sequences of siRNAs used in gene expression knockdown experiments.

siRNA	SN[a] sequence	ASN[a] sequence
siSTAT1	5'-ggauugaaagcauccuagaacucau-3'	5'-augaguucuaggaugcuuucaaucc-3'
siIFIH1	5'-cccucauuaucauugaugaauguca-3'	5'-ugacauucaucaaugauaaugaggg-3'
siRNA	5'-agcuacacuaucgagcaauuaacuu-3'	5'-aaguuaauugcucgauaguguagcu-3'

[a] SN, Sense; ASN, Antisense

Table S5. Sequences of primers used in SYBR® Green qRT-PCR experiments.

Primer	SN[a] sequence	ASN[a] sequence
STAT1	5'-ttcaggaagacccaatccag-3'	5'-cccgactgagcctgattaaa-3'
IFIH1	5'-agtctggcaccttggttgga-3'	5'-caaacgatggagagggcaag-3'
HMBS	5'-ggcaatgcggctgcaa-3'	5'-gggtacccacgcgaatcac-3'

[a] SN, Sense; ASN, Antisense

Supplementary Figures

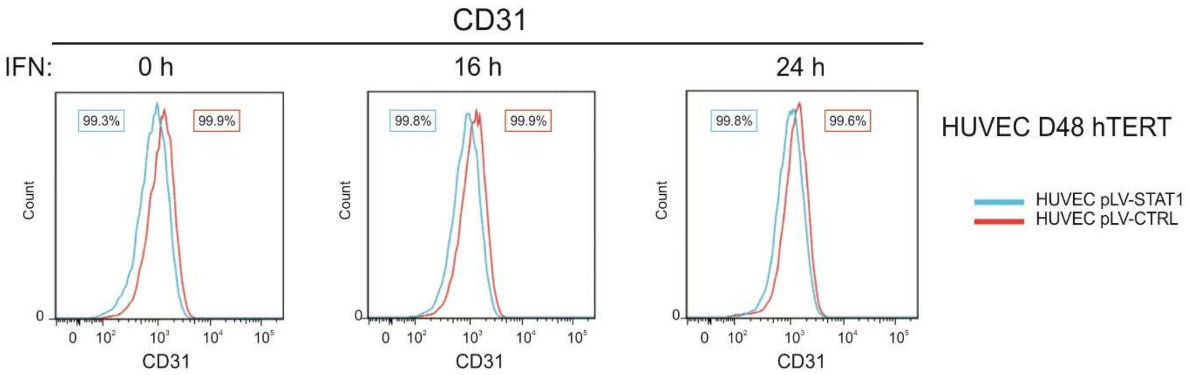

Figure S1. Effects of STAT1 overexpression on CD31. Flow cytometric analysis of HUVEC cells transduced with a lentiviral vector coding wild-type STAT1 cDNA and control. STAT1 overexpression mildly downregulated intensity of CD31 in a single cell culture (HUVEC D48) but did not affect the percentage of positive cells. Differently from STAT1 downregulation on IFNAR1, down-regulation in CD31 intensity is detected also in absence of IFN-α stimulation. CD31 expression was evaluated relative to isotype control (not shown).
